# Ebola virus: from in-situ simulation to SOP development

**DOI:** 10.1186/1757-7241-23-S2-A25

**Published:** 2015-09-11

**Authors:** Per P Bredmose, Gunnar Farstad, Odd Gunnar Paulsen, Marit E Hetland, Stephen Sollid

**Affiliations:** 1 Norwegian Air Ambulance Foundation, Drøbak, Norway; 2 Air Ambulance department, Prehospital Centre, Oslo University Hospital, Norway

## 

Pre-hospital care (PHC) personnel can be exposed to patients with infections at any time. The recent epidemic of Ebola hemorrhagic fever has highlighted the need for guidelines (SOPs) and competence in handling patients with infectious diseases in a safe manner, for both patient and PHC crew. Low fidelity in situ simulation can be an effective tool for training crews and developing SOPs.

## Methods

Three on call Helicopter Emergency Medical Service (HEMS) crews (HEMS physician, HEMS crew member and pilot) participated in a simulation exercise on management of a patient with potential symptoms of Ebola virus disease. A HEMS physician trained as a simulation training facilitator facilitated the simulation. Goals for the simulation exercises were: correct management of the patient, correct use of personal protection equipment (PPE), and team safety on scene. The HEMS crew provided feedback after the training on a standardised feedback form with closed questions using a 7 point Likert scale. During the debriefing the facilitator recorded important learning points that could be used to improve SOPs.

## Results

All crewmembers provided feedback after the training. All reported high degrees of satisfaction and realism within the simulation on a 7-pt. Likert scale. A total of 12 points of potential danger and the need for focused training were identified.

This resulted in the development of an improved SOP in the department. The teams involved agreed that simulation was a more efficient training method than traditional “PPE on/PPE off” training.

## Conclusion

Low fidelity simulation with the on call HEMS crew is an effective way to combine relevant training with the development and improvement of SOPs in an area where there is little clinical experience.

